# Impact on Practice with the Advisor™ HD Grid Mapping Catheter, Sensor Enabled™

**DOI:** 10.19102/icrm.2021.120125S

**Published:** 2021-01-15

This commentary roundtable offers insights from experts on how Advisor™ HD Grid Mapping Catheter, Sensor Enabled™ has impacted their practice, including challenges it has helped to overcome and contexts in which its use has proved particularly valuable.


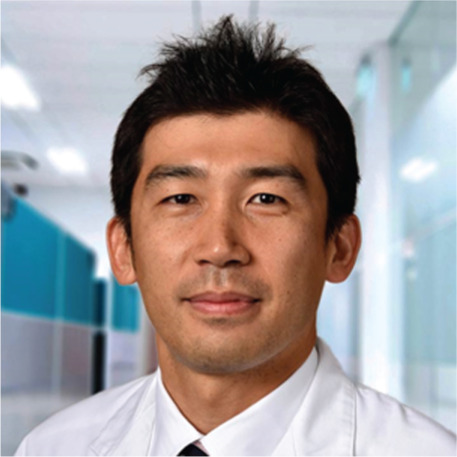
**Toshimasa Okabe, MD** The Ohio State University Wexner Medical Center, Columbus, OH, USA Dr. Okabe has received honoraria and consulting fees from Medtronic, Biosense Webster, and Abbott.
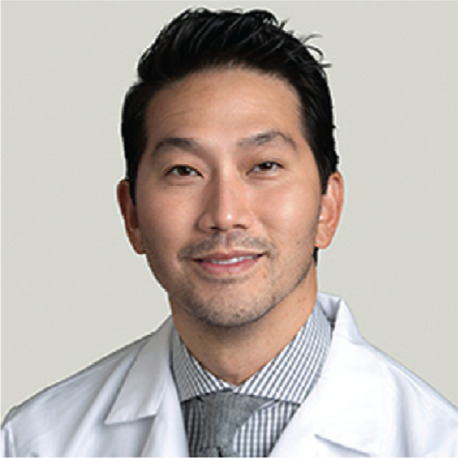
**Roderick Tung, MD** Pritzker School of Medicine, University of Chicago, Chicago, IL, USA Dr. Tung reports receiving honoraria for speaking from Abbott.
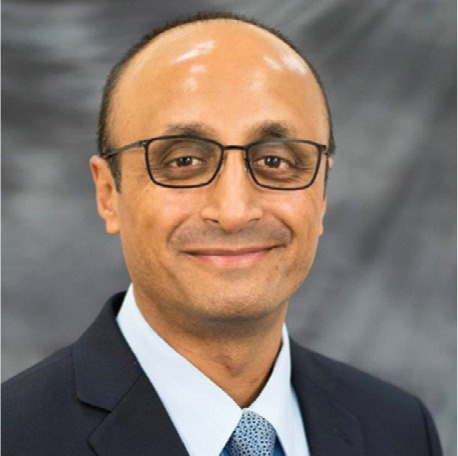
**Ashit Patel, MD** Cascade Cardiology LLC, Salem, OR, USA Dr. Patel reports performing consultative work for Abbott and Spectranetics.
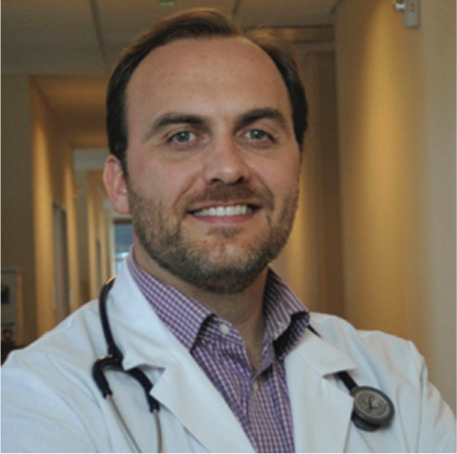
**Christopher Woods, MD** Sutter Health, Sacramento, CA, USA Dr. Woods is an unpaid consultant to Abbott.

## 1. What past procedural challenges has high-density grid technology enabled you to overcome?

*Dr. Okabe:* I have found that gaps in the pulmonary vein isolation (PVI) lines can be hard to find using conventional circular mapping catheters, which may result in redundant or unnecessary ablation applications. By carefully moving the Advisor™ HD Grid catheter along the lesion line and over the carina, any gaps can be readily revealed and targeted with focal ablation to close them.

*Dr. Patel:* The Advisor™ HD Grid mapping catheter has been a welcome addition to my practice and is now the main mapping catheter used in almost all complex cases. For the first time, it provides us with significant feedback concerning the contact force applied to intra-cardiac structures as suggested by the grid perturbation angle. This allows us to visualize and create actual intracavitary structures and to assure appropriate three-dimensional geometry. The catheter itself is quite flexible and atraumatic but, importantly, its design enables better maneuverabilitynear prosthetic valves or chorde. The EP field has not seen this before in a diagnostic catheter or high-density grid mapping catheter. From a recording standpoint, the Advisor™ HD Grid catheter has the ability to create orthogonal signal cross-check recordings, thus eliminating conduction directionality recording issues.

*Dr. Tung:* The Advisor™ HD Grid technology allows for evenly spaced mapping density in a small region. Contact in the chamber of interest has also been an important variable and this catheter allows for nice apposition with the endocardial or epicardium surface.Meanwhile, the redundancy in catheter bipoles facilitates confirmation that abnormal electrograms are useful and the directionality of the wavefront is an important area of further research.

*Dr. Woods:* The era of super–high-density mapping is obviously upon us. When I started in the field of cardiac electrophysiology (EP), not that long ago, we considered the achievement of a 200-point map to be a heroic feat and, really, a full day of mapping was required to obtain this kind of result! We did not even consider the concept of high-density mapping during training and, at the time, I had received a small business grant through the National Institutes of Health (NIH) to develop optical mapping technology to fill the niche now occupied by high-density mapping. Then, in the last decade, work across many centers has taught us the value of mapping density and, so, the era of high-density mapping began. Just a few years later, it has become routine to make super–high-density maps; I created an 80,000-point substrate map in less than one hour with the Advisor™ HD Grid Mapping Catheter, Sensor Enabled™ and EnSite Precision™ mapping system—so-called super–high-density mapping—for a ventricular tachycardia at 260 bpm as part of a routine day. While my NIH grant is history, the good news is that the advances in super–high-density mapping are very much present and have become foundational to a new era of EP.

I think the use of the Advisor™ HD Grid catheter in combination with the EnSite Precision™ mapping system has been a major reason for these advances. The Advisor™ HD Grid catheter is a superb catheter. It has top-of-the-class maneuverability and can easily display contact based on deformation and deflection on the electroanatomic map (or through fluoroscopy) to define contact. It is incredibly safe, which allows the operator to develop the confidence to reach everywhere one needs to in the heart safely. The fixed grid creates organized vectors of electrodes, which provide real-time feedback of circuits during mapping. To use super–high-density mapping, one has to adjust to allowing the computer to make the map. EnSite Precision™—and the forthcoming EnsiteX system—do just that with aplomb.

However, we are still EPs and being able to adjudicate signals in real time is critical to analyzing and confirming the map. Thankfully, the Advisor™ HD Grid technology allows for this—and, in fact, was built for this—through its fixed structure of bipoles. I find this characteristic critical to my analysis of isochronal late-activation mapping and critical substrate mapping. Super–high-density mapping makes it challenging to analyze the map in a post-hoc fashion because of the high number of points. So, receiving this real-time feedback is crucial in my view.

## 2. In which cases/contexts do you find the employment of high-density grid technology to be of greatest benefit in and why?

*Dr. Okabe:* The use of the Advisor™ HD Grid catheter has been particularly valuable in enabling rapid localization of gaps in prior ablation lines in redo atrial fibrillation cases and in identifying reentrant circuits in the setting of post-PVI atrial tachycardia. Additionally, as compared with when using a point-by-point mapping technique, substrate mapping (bipolar voltage and identification of late potentials) using the Advisor™ HD Grid catheter is efficient and able to pick up electrograms not seen when using an ablation catheter during the ablation of scar ventricular tachycardia.

*Dr. Patel:* Although the Advisor™ HD Grid catheter can be used in mapping virtually any chamber or structure, I find the most benefit exists when mapping intracavitary structures in terms of attaining more accurate anatomical reconstruction, high-density signals, and the ability to see the directionality of conduction. The Advisor™ HD Grid catheter has made it easier to evaluate moderator bands and papillary muscle in ventricular tachycardia/premature ventricular complex ablation cases. It is also a very resilient catheter when used to map the coronary cusps and subaortic valve structures.Given the design of the catheter, I believe it would be equally effective in epicardial cases.

*Dr. Tung:* The Advisor™ HD Grid catheter is one of the few catheters that maps that basal portion of the ventricle better than mid- or apical segments.We have found it particularly valuable to map the septum, left-sided conduction system, and periaortic region underneath the aortic valve.It has given us the ability to demonstrate small, localized reentrant mechanisms in patients with anteroseptal nonischemic cardiomyopathy substrates.^1^

*Dr. Woods:* I think, and data has shown, that super–high-density mapping, such as with the Advisor™ HD Grid catheter, provides the most usable data within substrate, where it has been proven to provide better delineation of circuits with more usable data.^2^ Most critically, one not only sees normal tissue better but can also visualize scar better. The entire field of functional scar analysis is possible because of high-density mapping; routinely, we observe signals with the Advisor™ HD Grid catheter that a mapping catheter is fully blind to. I also think that the ability of EnSite Precision™ to employ a dynamic window of interest, dynamic voltage settings, and isochronal late-activation mapping (ILAM) are critical features for this analysis. This has proved useful as demonstrated by Roderick Tung’s group at the University of Chicago, for example, as part of ILAM.^3^ We have seen the same benefit in delineating critical areas in substrate to maintain atrial flutters (submitted). Furthermore, I have also had tremendous success with managing far more straightforward arrhythmias like Wolff–Parkinson–White. Our group’s description of open-window mapping bore this out.^4^ I love this technique because, together with revealing the beauty of arrhythmia circuits, it demonstrates the ability of high-density mapping and computation to fully automate ablation targets accurately, which foreshadows where we are going. What an era of EP to be part of!
